# A Novel Haptic Cardiac Simulator: Mixed Methods Pilot Evaluation in Medical Students and Educators

**DOI:** 10.2196/83199

**Published:** 2026-05-15

**Authors:** Samuel James House, Robert Jay, David Graham O'Brien

**Affiliations:** 1 NHS Tayside Dundee United Kingdom; 2 College of Health and Science University of Lincoln Lincoln United Kingdom; 3 School of Medicine University of Nottingham Nottingham United Kingdom

**Keywords:** medical education, cardiac auscultation, haptic technology, multisensory learning, mobile health, mobile apps, smartphone, computer simulation, heart sounds, heart murmurs, educational technology, self-directed learning, physical examination, vibration, feedback

## Abstract

**Background:**

Cardiac auscultation is an essential component of clinical examination but is often challenging to achieve proficiency in. Self-contained, multisensory learning resources that incorporate simultaneous visual and haptic stimuli offer a unique approach to supporting learners in acquiring this core skill.

**Objective:**

This pilot study of both medical students and clinical educators evaluated the utility of a novel iPhone app, Haptic Heart, which generates haptic vibrations to simulate heart sounds and murmurs. We aimed to explore the perceptions of students and educators when using haptics as a learning resource and the underlying reasons behind these perceptions and to gather lessons that would inform future development of the resource.

**Methods:**

Clinical-year medical students from the Lincoln Medical School with access to an iPhone were invited to trial Haptic Heart between October 2023 and December 2024. Cardiology specialists involved in clinical education were also invited to take part. After using the app, participants were asked to complete a modified version of the 12-item Evaluation of Technology-Enhanced Learning Materials: Learner Perceptions questionnaire that included additional free-text items. Educators were also asked to comment on the resource’s authenticity and perceived usefulness. Quantitative responses were analyzed using descriptive statistics; free-text responses were analyzed for common themes.

**Results:**

A total of 21 students and 18 educators completed the evaluation. Both cohorts returned positive responses across nearly all questionnaire items, with students showing near universal agreement that the app was of excellent quality (21/21, 100%), supported their learning needs (21/21, 100%), and would change their clinical practice (20/21, 95.2%). Educators similarly rated the resource highly for learning utility (16/18, 88.9%) and authenticity (13/18, 72.2%). Reported technical difficulties were minimal for students (1/21, 4.8%) and educators (2/18, 11.1%). Analysis of free-text responses suggested that learners valued the ability to “feel” murmurs and to vary heart rate. Educators highlighted the resource’s novelty and innovation, although some noted concerns about audio quality when using a stethoscope to auscultate haptic vibrations directly.

**Conclusions:**

This pilot evaluation demonstrates the potential of smartphone-based haptic technology as a tool for medical education. Haptic Heart was perceived by both students and educators as an innovative educational tool for cardiac auscultation. Further work should focus on expanding the range of haptic patterns provided and exploring the effectiveness of these resources on learning.

## Introduction

Cardiac auscultation is an essential skill for cardiovascular examination and is taught in undergraduate medical curricula worldwide. Despite this, it remains an area that many medical students and resident physicians find challenging [1,2]. The rapid progression of mobile technology has created unprecedented opportunities for innovation within medical education. Smartphones are now near ubiquitous among clinicians and learners [3,4] and offer a powerful platform for self-contained, readily accessible learning resources. However, existing smartphone apps are predominantly audiovisual in approach, with a focus on communication, medical information, and clinical decision-making [5], failing to exploit their full multisensory potential.

Haptic technology provides tactile feedback through vibration or force and is most commonly used in medical education to enhance realism in tasks with inherent tactile elements, such as suturing or laparoscopic procedures [6,7]. However, multisensory learning techniques appear to improve memory recall more generally [8,9], and thus, using a multisensory approach when providing educational content may improve knowledge and recall [10].

Patrizio et al [11] recently explored the role of haptics in medical education focusing on teaching a concept rather than simulating the task’s inherent tactile component in cardiac auscultation. They found that introducing synchronized audio, visual, and haptic stimuli improved both knowledge retention and diagnostic accuracy compared to traditional teaching methods. While the haptic stimulation in this case was relatively primitive, it supported the concept that a multisensory learning approach may enhance the educational effectiveness of cardiac auscultation training.

To our knowledge, there has never been an attempt to use the haptic capabilities of smartphones as a medical teaching tool. On the basis of this, we developed Haptic Heart to explore the potential of a smartphone app that incorporates haptic and visual feedback as a combined learning resource to help medical students improve their cardiac auscultation skills.

## Methods

### Overview

**Figure 1:**
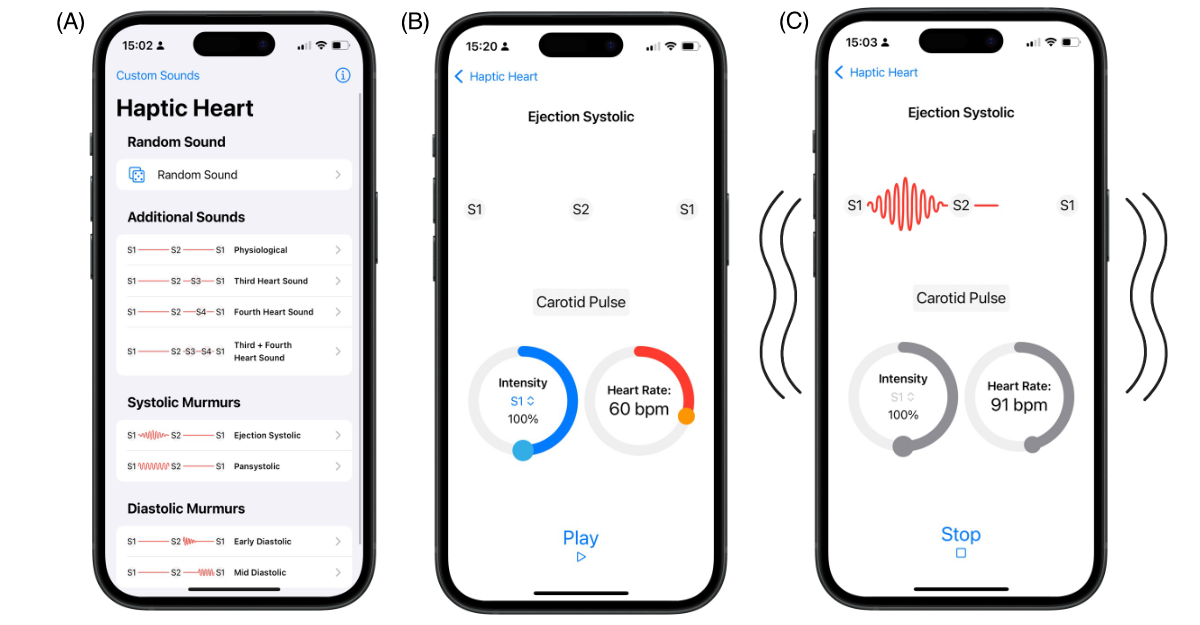
Initial screen of Haptic Heart showing the list of haptic patterns that can be selected (A), selected pattern screen showing the ability to alter the heart rate and intensity of the elements (B), and pattern-playing screen with simplified phonocardiogram and visual representation of the device vibrating (C).

The smartphone app was developed specifically for iPhone (Apple Inc) by using Apple’s Core Haptics framework as Android and similar platforms lack the necessary hardware and software to support the required haptic capabilities [12]. Multiple haptic patterns were used to mimic heart sounds S1 and S2, with the choice of including additional heart sounds (S3 and S4), ejection systolic, pansystolic, early diastolic, and middiastolic murmurs. The user can select the haptic pattern they wish to experience from the list of available options (Figure 1A).

Once the user has selected a pattern, they are then able to alter the heart rate and the intensity of any element within that pattern using the inbuilt sliders (Figure 1B). The app uses a mathematical model to dynamically determine the pattern, which is then generated by the iPhone’s Taptic Engine, with the user experiencing the device vibrate. A synchronized visual phonocardiogram is also displayed to the user, allowing them to both see and feel the haptic pattern selected (Figure 1C).

**Figure 2:**
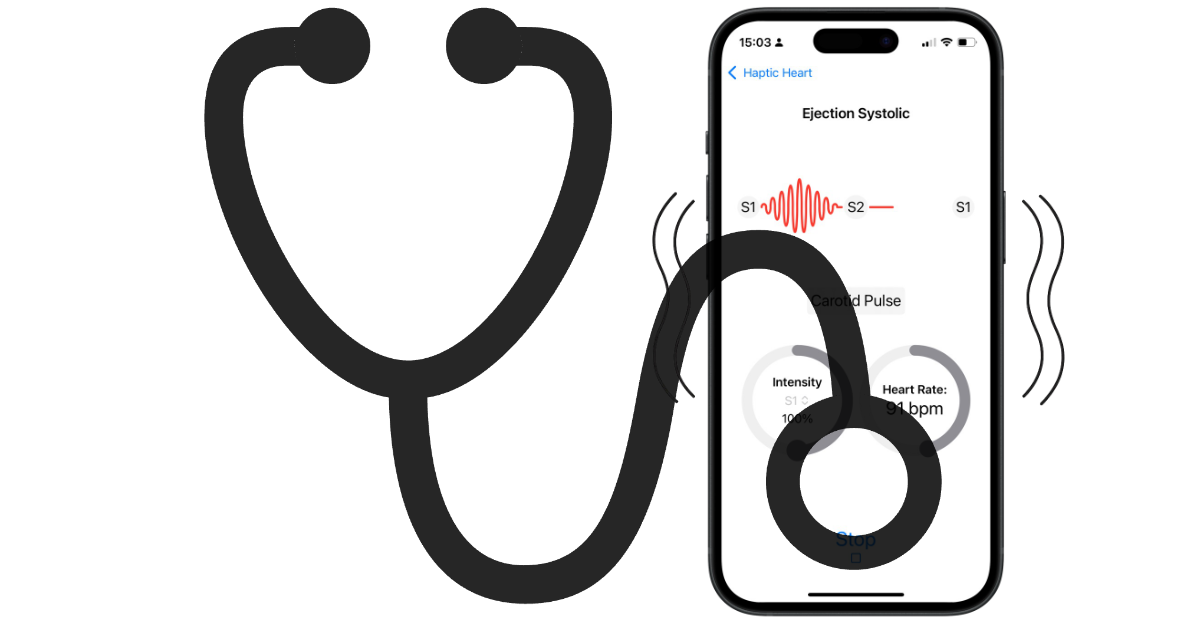
Graphical representation of how it is also possible to directly auscultate the iPhone to provide simultaneous haptic and auditory feedback by exploiting the sounds produced by the haptic motor as a byproduct of haptic generation.

The user is also able to place a stethoscope directly onto the iPhone, allowing for simultaneous auscultation while the haptic pattern is playing to generate auditory feedback (Figure 2). No audio files are currently used on the app, with all sounds being generated entirely by haptics created from the iPhone’s motor.

**Figure 3:**
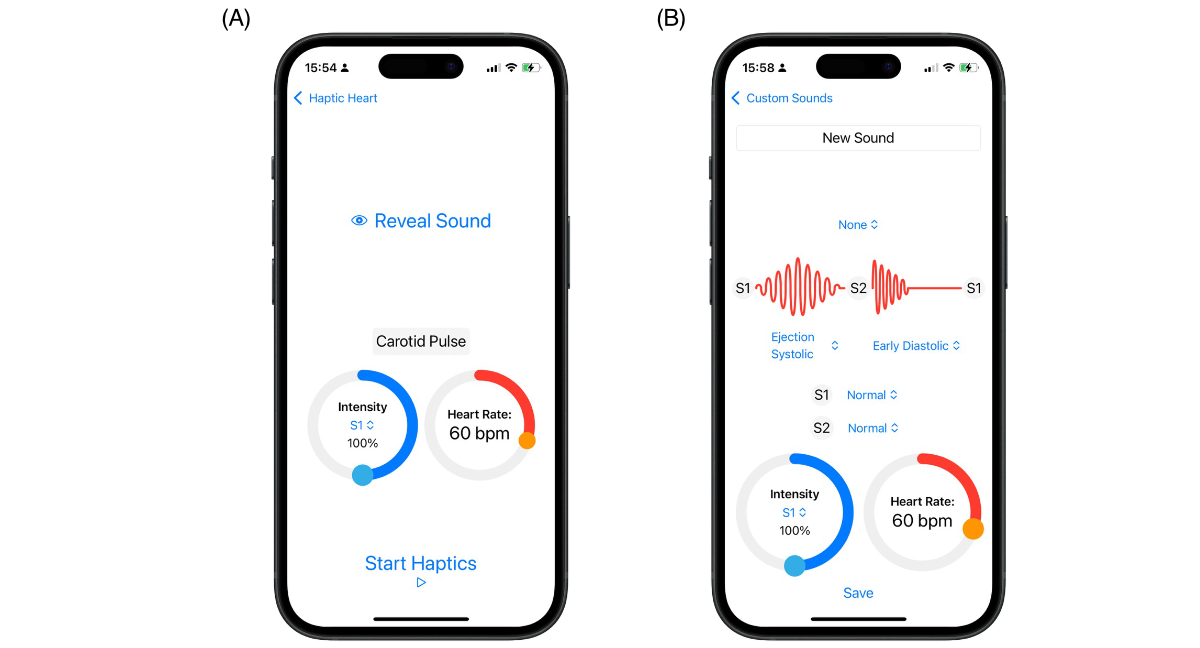
Haptic Heart quiz mode showing how the identity of the haptic pattern is kept hidden by hiding the name and phonocardiogram (A) and Haptic Heart custom sound mode showing how different haptic elements can be combined to make custom patterns (B).

The app also features a quiz function that presents the user with a random sound, which they must identify (Figure 3A). It is also possible to create custom patterns by combining different heart sounds and murmurs (Figure 3B).

Given this innovative concept, a pilot study was conducted to evaluate the user experience of this resource, loosely based on a framework designed to explore app feasibility, to determine whether to pursue further iterations and, if so, how best to develop them [13]. We aimed to explore the perceptions of students and educators when using haptics as a learning resource to complement physical cardiovascular system examination and the underlying reasons behind these perceptions [14] and to gather lessons that would inform the development of the resource and further research.

Between October 2023 to December 2024 a purposeful sample of medical students in their clinical years of study at Lincoln Medical School with access to an iPhone was invited to participate via posters displayed at their clinical teaching sites in addition to emails from course administrators. The posters and emails contained a QR code or hyperlink, respectively, which took the students to the study website, where they were able to read further about the resource before deciding whether to take part. Medical students were the target study population as the ability to both conduct “an appropriate physical examination” and “interpret findings from history, physical and mental state examinations” is an essential requirement for graduation as defined by the UK General Medical Council [15]. They are also a group that often historically underperforms in this area [16], making them an ideal target audience for future similar learning resources. Local cardiology specialist educators, again with access to an iPhone, were recruited either in person or via digital communication and selected for their expertise in cardiac examination, enabling them to evaluate Haptic Heart for realism and compare it with existing learning resources. After completing the online consent form, students and educators were given access to the app via a download link. After using the app, they were required to complete a questionnaire through pop-up prompts directing them to a Microsoft Form.

Both cohorts were asked to complete the short version of the Evaluation of Technology-Enhanced Learning Materials: Learner Perceptions questionnaire as originally described by Cook and Ellaway [17]. This was modified to remove questions irrelevant to the resource, leaving 12 statements all assessed using a 7-point Likert scale. An option to leave free-text comments for each question was also added to help explore the reasons behind the participants’ perceptions. Both cohorts were also asked open-ended questions about the quality of the app, how it could be improved, and which element most contributed to their engagement as learners. The educator cohort was additionally asked to comment on the authenticity of the app’s representation of heart sounds and on its perceived usefulness for students’ learning. The questionnaire completed by the students and educators are included in Multimedia Appendices 1 and 2, respectively.

The quantitative responses were analyzed using descriptive statistics. The 7-point Likert scale was converted to numerical scores (range −3 to 3; 0=neutral, +3=strong agreement, and −3=strong disagreement) to facilitate graphical representation. Free-text responses were reviewed, and common themes were identified regarding the use of haptics as a learning tool, perceptions of the app more generally, and suggestions for future improvement.

### Ethical Considerations

This study was approved by the University of Nottingham’s Faculty of Medicine and Health Sciences Research Ethics Committee (reference: FMHS 376-0923). This study was performed in line with the principles of the Declaration of Helsinki and followed the principles of voluntary participation, confidentiality, and no harm. Participants were informed that their participation was entirely voluntary and that they had the right to withdraw from the study at any time without providing a reason and without any negative consequences for their future studies or employment. All participants provided either oral or written informed consent for taking part. All data were anonymized at the time of collection. Participants were not compensated for their participation in this research.

## Results

### Data Analysis

**Figure 4:**
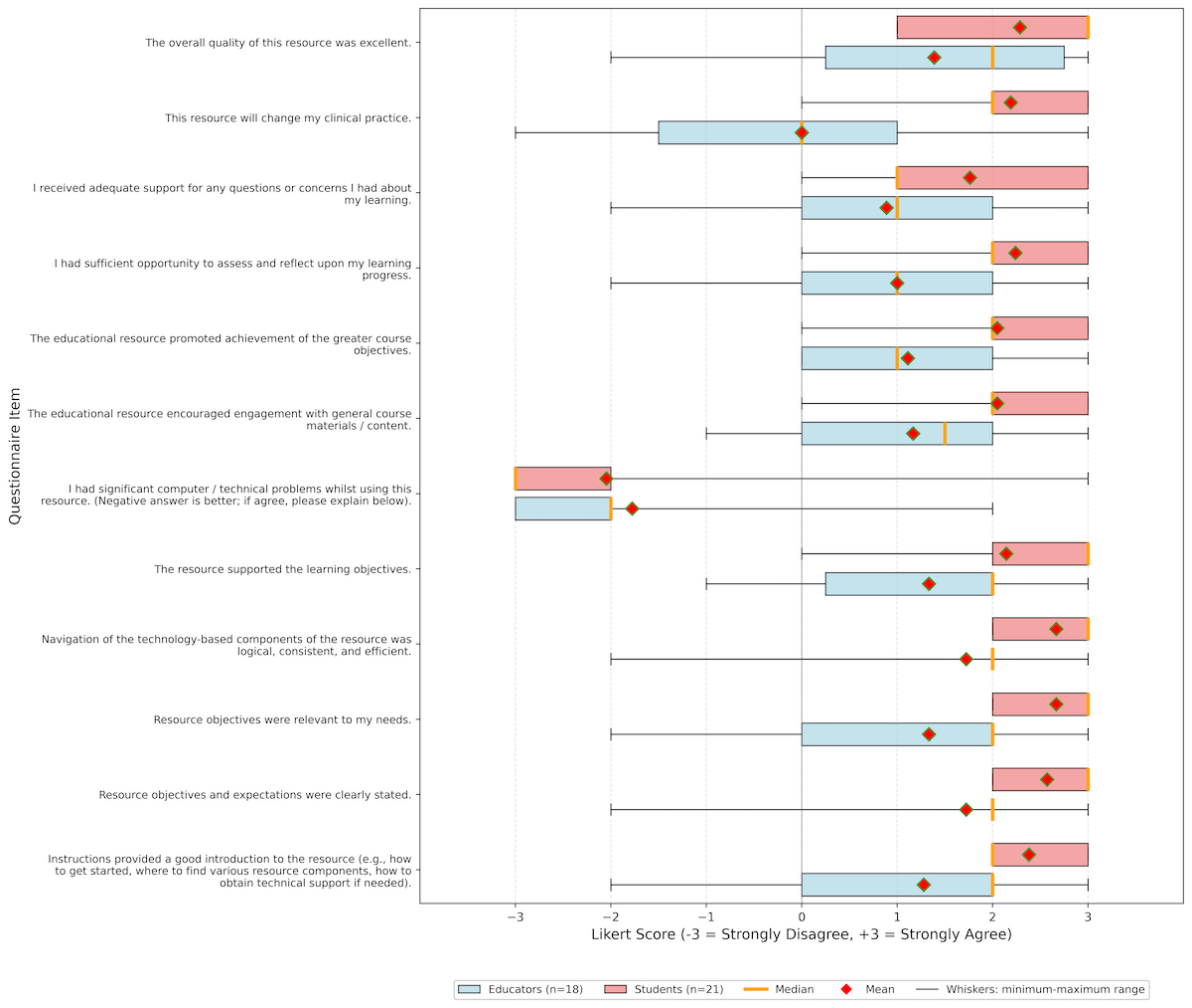
Box plot comparing Evaluation of Technology-Enhanced Learning Materials: Learner Perceptions questionnaire scores between medical students (n=21) and cardiology educators (n=18). Responses were provided on a 7-point Likert scale (−3=“strongly disagree,” 0=“neutral,” and 3=“strongly agree”). Higher scores indicate more positive responses except for the “technical problems” question, where lower scores are favorable.

A total of 21 medical students and 18 educators completed the questionnaire. Free-text comments were provided by 28.6% (6/21) of the students and all educators (18/18, 100%). Both student and educator groups provided positive responses across nearly all items in the Evaluation of Technology-Enhanced Learning Materials: Learner Perceptions questionnaire (Figure 4). Among the student group, there was universal agreement that the app’s objectives were relevant to their learning needs and that the app’s quality was excellent (21/21,100%). All but one student (20/21, 95.2%) agreed that the app would change their clinical practice. There was more variation in responses regarding the app’s learning objectives. When asked whether the app supported these objectives, 76.2% (16/21) of the students agreed. Similarly, 85.7% (18/21) of the students reported that Haptic Heart promoted achievement of the course objectives by improving cardiovascular examination skills.

Among educators, the evaluation of the app was also positive, with most agreeing that the overall quality of the resource was excellent (13/18, 72.2%) and that navigation of the technology-based components of the resource was logical, consistent, and efficient (16/18, 88.9%). Similar to the student cohort, most agreed that the app supported the learning objectives (13/18, 72.2%) and promoted the achievement of the greater course objectives (10/18, 55.6%). Responses to the educator-only questions were also positive, with nearly unanimous agreement that the resource was useful for learning (16/18, 88.9%), with 72.2% (13/18) feeling that the app provided an authentic representation of clinical findings.

There were minimal technical difficulties, with 4.8% (1/21) of the students and 11.1% (2/18) of the educators reporting issues in using the resource.

### Free-Text Response Themes

The free-text responses highlighted the potential value of haptics as a learning resource. Students particularly valued the tactile experience, with one noting that it was “really useful to be able to feel and listen to the murmurs at different heart rates” and another stating that “being able to feel the vibrations through the device but also auscultate was so cool.” The ability to “feel” the murmurs provided an advantage in helping them learn in preparation for assessments.

Comments from the educators indicated that the use of haptics was a novel and innovative way to integrate smartphone technology into teaching. One respondent described it as a “very interesting and well thought-out resource which I think will be of huge help to medical students and foundation doctors.” Haptic feedback was reported as particularly useful for teaching the timing and patterns of different heart sounds and murmurs, with several educators expressing interest in incorporating haptic feedback into regular teaching. However, some concerns were raised regarding the quality of the audio generated as a by-product of the haptic motor when using a stethoscope to directly auscultate the device to incorporate auditory feedback.

Students valued being able to alter the heart rate as this enhanced real-world application, with one student reporting the following:

It’s really useful to be able to feel and listen to murmurs at different heart rates.

Other feedback emphasized the advantages of practicing independently outside the clinical setting and using self-test questions. The educators also found that the ability to alter the heart rate and explore its impact on the pattern was beneficial. They also noted how displaying the synchronized phonocardiogram while the haptic pattern was running was helpful. The resource’s smartphone app format made it both easily accessible and convenient for bedside use.

Suggestions from both groups included expanding the range of murmurs and heart sounds within the app, such as the pattern of severe aortic stenosis incorporating a quiet S2 sounds to demonstrate increased severity or including metallic heart sounds. Educators also suggested that educational information on the clinical significance and relevance of the simulated findings be included. Students requested verbal descriptions of what they were experiencing to help support their interpretations:

Personally, I would have liked some verbal descriptions of the murmur just to keep memory fresh when describing the murmur to others, as in using correct terminology.

The educator cohort also noted that the audio was insufficiently realistic when auscultating the device using a stethoscope, with one educator reporting the following:

...it didn’t exactly mimic the pitch of an ESM [ejection systolic murmur] or pan-systolic murmur (I appreciate that it’s artificial and doesn’t replace actual bedside findings).

Another educator commented the following:

I could not hear any audio through my device and had to place my ear next to the speakers (despite max volume), despite which the sound was not realistic and very faint.

An additional educator also commented the following:

Unfortunately it sounded very tin-like, metallic and artificial on my phone. (iPhone 15 pro max) and I was using a Littman Cardiology III stage scope on the phone to be realistic.

## Discussion

### Principal Findings

To our knowledge, this is the first attempt to use smartphone haptic motors to support cardiac examination techniques; therefore, Haptic Heart represents a novel resource. This pilot evaluation emphasizes the potential of such an app, with an almost universally positive response from students and educators. As with all new interventions, there is a risk of a “novelty effect,” whereby the positive reception may partly reflect participants’ enthusiasm for new technology rather than any actual educational effectiveness [18].

However, several factors suggest that our findings extend beyond mere novelty. Free-text comments from students indicate that they specifically valued the ability to “feel” the murmurs. Similarly, educators repeatedly noted that the use of haptics in this context was beneficial. The students’ comments also suggest that they found haptics beneficial for training, particularly in preparation for clinical assessments. These findings are consistent with those of the existing literature, where multisensory approaches incorporating simultaneous visual and auditory stimuli have been shown to increase recognition rates compared to a single approach [8,9,19]. These findings, taken alongside those of Patrizio et al [11], suggest that there may be real benefit in using haptics in medical education, which is something demanding further exploration.

It is also important to consider the impact of delivering haptics through a smartphone app as this was itself a novel approach. The smartphone’s supporting features, such as its inherent portability, the option to use it at the bedside, and the ability to dynamically create the pattern based on heart rate and alter it, all appeared beneficial.

There was an overall difference in perception between educators and students, with the latter rating the app more positively across all 12 questions. Analysis of the educators’ free-text comments reveals a perception of isolation of the resource from the clinical context due to the minimal information provided with regard to the clinical relevance of different findings. The students’ responses, on the other hand, reveal that they were more focused on practicing the technical elements of interpretation, such as different heart rates, suggesting a different perception of the usefulness of the app. Several comments from the educators on potential improvements focused on this common theme, including suggestions for additional information on determining the severity of a valve lesion based on cardiac auscultation and demonstrating the changes in heart sounds and murmurs that occur with increased severity. Other suggested improvements from both groups shared a common theme of broadening the range of available heart sounds and murmurs available on the resource.

Another area for improvement highlighted by educators was audio quality, described as “tin-like, metallic and artificial.” However, analysis of the free-text comments reveals a possible misunderstanding of the technology among some of the educators who took part. For example, one educator reported that they “could not hear any audio through my device and had to place my ear next to the speakers (despite max volume) despite which the sound was not realistic and very faint.” This suggests that several of the educators did not recognize the key innovation (ie, the haptic element of the app), instead considering this an audio resource requiring auscultation. When comparing responses from those who highlighted sound quality with responses from those who did not, the former were more likely to rate the app lower, and those who appreciated the haptic component were also more likely to comment on the novelty and innovative nature of the combined approach. This demonstrates a limitation in our evaluation of the resource. The use of a stethoscope to auscultate the device was intended as a secondary feature, not the primary one; this should be made clearer to users of the app and probably requires greater scaffolding for how to effectively use the resource.

To contextualize these findings, it is also important to compare Haptic Heart with existing resources. Garvick et al [20] showed that physician associate students using Littmann Learning, a mobile app containing real recorded heart sounds, achieved significantly higher clinical knowledge scores than those not using the app. While the goal of Haptic Heart is the same (ie, to improve students’ cardiac auscultation skills), the approach is fundamentally different. While both resources benefit from the inherent portability of a smartphone platform, Haptic Heart was designed to provide a multisensory approach to understanding different heart sounds and murmurs via haptics and concurrent visual feedback. The positive reception from both students and educators suggests that using haptics in this way has potential educational value. It should be evaluated further in addition to and in comparison with existing purely auditory-based resources. Future research should directly compare learning outcomes between audio-only and multisensory, haptically enhanced approaches to determine whether the multisensory haptic approach provides any measurable educational benefits beyond those of traditional audio-based resources. In a similar vein, it may certainly be worth exploring whether it would be feasible to combine visual, haptic, and dedicated high-quality audio stimuli into a single resource.

Given that this was a pilot evaluation of a new resource, it has several inherent limitations. Our participants were recruited through purposeful, convenience sampling rather than systematic random sampling. Both cohorts of volunteers were possibly technology enthusiasts and were recruited from the same geographical region. Similarly, due to hardware and software limitations, the app was available only on the iPhone platform; thus, only those with an iPhone were recruited, further reducing the generalizability of our results. Furthermore, the size and, therefore, output of the haptic motor is nonstandardized across different iPhone models [12]. The presence of accessories such as phone cases may also affect how vibrations are perceived; unfortunately, we did not collect this information in this study. Our sample sizes for both cohorts were small, and the evaluation was conducted over a relatively short period. Finally, our evaluation focused on both students’ and educators’ perceptions, with no objective assessment of the app’s impact on the learning outcomes achieved.

### Conclusions

While haptics technologies are already in use in medical education, they are typically used to enhance task realism when there is an inherent tactile element. This pilot evaluation is, to our knowledge, the first attempt to explore smartphone-based haptic technology for cardiac examination training. This work demonstrates that developing a haptic-based smartphone app for this purpose is not only technically feasible but also engaging for learners and well received by educators. However, it is essential to formally evaluate the educational impact of this resource, including modifying the app’s instructional design to optimize its influence on learning performance and outcomes. Furthermore, it is important to evaluate Haptic Heart in comparison with existing audio-focused cardiac examination learning resources to better define what educational benefit arises from the multisensory haptic approach and whether further development is justifiable. Similarly, it may be worth exploring the benefits of combining visual, haptic, and dedicated high-quality audio stimuli into a single resource. Beyond this, there is a need to consider the broader potential of haptics in other related areas of medical education where they may be complementary and beneficial.

The app itself could be further enhanced by expanding the number of included haptic patterns and including information on the clinical significance of various heart sounds and murmurs. Future, more extensive evaluation may also help clarify how the resource should best be used.

## Data Availability

The datasets for students and educators are available for review in Multimedia Appendices 3 and 4, respectively.

## Appendix

Multimedia Appendix 1 The modified short version of the Evaluation of Technology-Enhanced Learning Materials: Learner Perceptions questionnaire as received by the students.

Multimedia Appendix 2 The modified short version of the Evaluation of Technology-Enhanced Learning Materials: Learner Perceptions questionnaire as received by the cardiology specialist educators, with additional questions on authenticity of clinical findings and usefulness for learning.

Multimedia Appendix 3 Dataset of student questionnaire responses.

Multimedia Appendix 4 Dataset of educator questionnaire responses.
