# Supplementary material for: A Novel Haptic Cardiac Simulator: Mixed Methods Pilot Evaluation in Medical Students and Educators
Source: JMIR Form Res. 2026 May 15;10:e83199. doi: 10.2196/83199 (PMC13221619; doi:10.2196/83199)
Supplement: Multimedia Appendix 1 [file formative_v10i1e83199_app1.docx]

| **Question** | **Answer Format** |
| --- | --- |
| 1. Year of Study | Stage of Study Tick box + Other Free Text option |
| 1. Please enter your email address (this will only be used to confirm participation and avoid sending you further reminders) | Free Text |
| 1. Instructions provided a good introduction to the resource (e.g., how to get started, where to find various resource components, how to obtain technical support if needed) | 7 Point Likert Response |
| 1. Additional Comments | Free Text |
| 1. Resource objectives and expectations were clearly stated | 7 Point Likert Response |
| 1. Additional Comments | Free Text |
| 1. Resource objectives were relevant to my needs | 7 Point Likert Response |
| 1. Additional Comments | Free Text |
| 1. Navigation of the technology-based components of the resource was logical, consistent, and efficient. | 7 Point Likert Response |
| 1. Additional Comments | Free Text |
| 1. The resource supported the learning objectives | 7 Point Likert Response |
| 1. Additional Comments | Free Text |
| 1. I had significant computer / technical problems whilst using this resource. (Negative answer is better; if agree, please explain below) | 7 Point Likert Response |
| 1. Additional Comments | Free Text |
| 1. The educational resource encouraged engagement with general course materials / content. | 7 Point Likert Response |
| 1. Additional Comments | Free Text |
| 1. The educational resource promoted achievement of the greater course objectives. | 7 Point Likert Response |
| 1. Additional Comments | Free Text |
| 1. I had sufficient opportunity to assess and reflect upon my learning progress. | 7 Point Likert Response |
| 1. Additional Comments | Free Text |
| 1. I received adequate support for any questions or concerns I had about my learning. | 7 Point Likert Response |
| 1. Additional Comments | Free Text |
| 1. This resource will change my clinical practice | 7 Point Likert Response |
| 1. Additional Comments | Free Text |
| 1. The overall quality of this resource was excellent | 7 Point Likert Response |
| 1. Additional Comments | Free Text |
| 1. How could the quality of the resource be improved? What would you keep the same? What would you change, remove, or add? Please describe and explain | Free Text |
| 1. Overall, what elements of this resource most contributed to your excitement and engagement as a learner? What could have been done to improve your engagement? Please describe and explain. | Free Text |
